# Enzymatic Synthesis of the Flavone Glucosides, Prunin and Isoquercetin, and the Aglycones, Naringenin and Quercetin, with Selective **α**-L-Rhamnosidase and **β**-D-Glucosidase Activities of Naringinase

**DOI:** 10.4061/2011/692618

**Published:** 2011-09-15

**Authors:** Hélder Vila-Real, António J. Alfaia, M. Rosário Bronze, António R. T. Calado, Maria H. L. Ribeiro

**Affiliations:** ^1^Research Institute for Medicines and Pharmaceutical Sciences (i-Med-UL), Faculty of Pharmacy, University of Lisbon, Avenue Prof. Gama Pinto, 1649-003 Lisbon, Portugal; ^2^Departmento de Química, Instituto de Tecnologia Química e Biológica, Apartado 127, Av. República, 2784-505 Oeiras, Portugal

## Abstract

The production of flavonoid glycosides by removing rhamnose from rutinosides can be accomplished through enzymatic catalysis. Naringinase is an enzyme complex, expressing both *α*-L-rhamnosidase and *β*-D-glucosidase activities, with application in glycosides hydrolysis. To produce monoglycosylated flavonoids with naringinase, the expression of *β*-D-glucosidase activity is not desirable leading to the need of expensive methods for *α*-L-rhamnosidase purification. Therefore, the main purpose of this study was the inactivation of *β*-D-glucosidase activity expressed by naringinase keeping *α*-L-rhamnosidase with a high retention activity. Response surface methodology (RSM) was used to evaluate the effects of temperature and pH on *β*-D-glucosidase inactivation. A selective inactivation of *β*-D-glucosidase activity of naringinase was achieved at 81.5°C and pH 3.9, keeping a very high residual activity of *α*-L-rhamnosidase (78%). This was a crucial achievement towards an easy and cheap production method of very expensive flavonoids, like prunin and isoquercetin starting from naringin and rutin, respectively.

## 1. Introduction

The production of monoglycosylated flavonoids is an interesting application field of enzymatic biocatalysis by removing the rhamnose radical, namely, of rutinosides, as well as in the production of rhamnose itself. The deglycosylation of flavonoids can improve biological activity through its bioavailability improvement [1]. Such improvement may be related not only to pharmacokinetics and pharmacodynamics, but also, to the overall molecule structure. This result supports that monoglycosylated flavonoids and its aglycones are more easily absorbed, than the original lead compound. Flavonoids show a wide range of beneficial effects on human health, including cardiovascular and chronic diseases and certain forms of cancer activity [2–4], as well as antimicrobial, antioxidant, antiviral, antiplatelet, anti-ischemic, antitumor, anti-inflammatory, antiallergic, estrogenic, and radical-scavenging activities [5, 6]. 

Quercetin, isoquercetin, and other flavonoids have been shown to modify eicosanoid biosynthesis (antiprostanoid and anti-inflammatory responses), protect low-density lipoprotein from oxidation (prevent atherosclerotic plaque formation), prevent platelet aggregation (antithrombic effects), and promote relaxation of cardiovascular smooth muscle (antihypertensive, antiarrhythmic effects) [6]. Most beneficial health effects of quercetin are related to absorption, metabolism, and bioavailability into the human body. In addition, flavonoids, like prunin, have been shown to have antiviral properties [5]. The activity of flavonoids as inhibitors of reverse transcriptase suggests a place for these compounds in the control of retrovirus infections, such as acquired immunodeficiency syndrome. Moreover, to specific effects the broad-modulating effects of flavonoids can serve as starting material for drug development. Due to these numerous properties and applications, flavonoids have gained growing interest.

Conventional chemical methods for the preparation of flavonoids and saponins usually produce side reactions. In this context enzymatic modification is advantageous due to selectivity and mildness of the reaction conditions.

**Figure 1:**
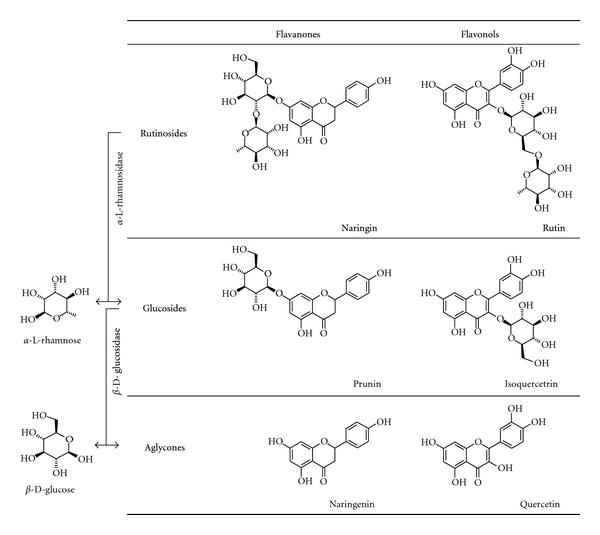
Scheme of the production of flavonoid glucosides and aglycones starting from its rutinosides precursors using an enzymatic approach (*α*-L-rhamnosidase and *β*-D-glucosidase activities of naringinase).

Naringinase is an enzyme complex used in compounds deglycosylation and with a high potential in food and pharmaceutical industries. Naringinase provides both *α*-L-rhamnosidase and *β*-D-glucosidase activities (Figure 1) and has been used to accomplish some glycosides hydrolysis [7–9].

Statistical design of experiments is a useful tool to provide experimental schemes where the parameters (factors) under study are combined at different levels to determine the influence of a particular factor on the response. Additionally, an adequate experimental design not only allows the determination of individual effects but also the interactions between them can be uncovered. 

Response surface methodology (RSM) is an efficient statistical technique for the modelling and optimization of multiple variables in order to predict the best performance conditions with a minimum number of experiments [10]. It consists of a group of mathematical and statistical procedures that can be used to study relationships between one or more responses and a number of independent variables. RSM defines the effect of the independent variables, alone or in combination, on the process. In addition, to analyze the effects of the independent variables, this experimental methodology generates a mathematical model that may accurately describe the overall process. These methods find major importance when the effect of one variable is affected by the setting of another. Such “interaction effects” between variables are difficult to detect by a traditional experimental setup where one variable is changed at a time. The coefficients of the mathematical model (usually a polynomial equation) representing the variations of the experimental response of interest may be evaluated with high precision. Additionally, RSM has the advantage of being less expensive and time consuming than the classical methods [11].

RSM is a nonconventional approach that has been successfully used for the optimization of enzymatic reactions conditions [11–13], medium composition [14, 15]. In this work, central composite rotatable design (CCRD) and RSM were used to compare the combined effects of temperature and pH on *β*-D-glucosidase activity of naringinase. 

To produce monoglycosylated flavonoids with naringinase, the presence of *β*-D-glucosidase activity expression leads to the need of using selective inhibitors or even expensive methods of *α*-L-rhamnosidase purification [16]. In the current study the effect of pH and temperature on *β*-D-glucosidase inactivation from naringinase were evaluated using a central composite face-centered design. It was possible to define the best factor combination of *β*-D-glucosidase inactivation from naringinase, keeping *α*-L-rhamnosidase expression in a high activity. This naringinase with inactivated *β*-D-glucosidase and expressing *α*-L-rhamnosidase allowed the production of two very expensive flavonoid glucosides, prunin and isoquercetin, in an easy and cheap bioprocess starting from naringin and rutin, respectively.

## 2. Materials and Methods

### 2.1. Chemicals and Enzyme

*p*-Nitrophenyl *α*-L-rhamnopyranoside (4-NRP) and *p*-nitrophenyl *β*-D-glucopyranoside (4-NGP) were from Sigma-Aldrich. All other chemicals were of reagent grade and were obtained from various sources.

Naringinase (CAS no. 9068-31-9, cat. no. 1385) from *Penicillium decumbens *was obtained from Sigma-Aldrich and stored at −20°C. The lyophilized naringinase powder was dissolved in the appropriate buffer solution 24 hours before experiments and was kept at 4°C.

### 2.2. Analytical Methods

The concentration of *p*-nitrophenol produced after the hydrolysis of 4-NRP and 4-NGP was evaluated spectrophotometrically (*Zenith 3100* spectrofluorimeter) at *λ* = 340 nm, using a calibration curve of each compound.

The flavonoid rutinosides, glucosides, and aglycones were eluted running a TLC on an RP-18 silica-gel plate with methanol-water-acetic acid (50/44/6, v/v/v) [17]. The spot visualization was evaluated under UV light at 254 nm followed by spraying with a freshly prepared solution of acetic acid/sulphuric acid concentrated/*p*-anisaldehyde (100/2/1, v/v/v) heated at 150°C for 5 min [18].

The HPLC-DAD-ESI-MS/MS experiments used to identify the produced compounds (prunin, naringenin, isoquercetin, and quercetin) were performed with a liquid chromatograph (Alliance, Waters 2695 Separation Module) system with a photodiode array detector (DAD, Waters 2996) set at 280 nm (for monitoring) in tandem with a mass spectrometer (Micromass Quattro Micro API) with a Triple Quadrupole (TQ) and an electrospray ion source (ESI) operating in negative mode. Chromatographic conditions were as follows: column C18 (Synergi, Phenomenex) 100 mm × 2.0 mm, 2.5 *μ*m; eluent (A) water-formic acid (99.5 : 0.5, v/v), (B) acetonitrile (LC-MS grade, Merck). The linear gradient was at initial time 95% eluent A, and at 30 min 60% eluent A, at 45 min 10% eluent A. The flow rate was 0.25 mL/min and the column temperature 35°C. Mass range was measured from 100–1000 amu. The ESI source conditions were adjusted as follows: source capillary operating at 2.5 kV and the extraction cone at 30 V; the source temperature was 150°C and the desolvation temperature was 350°C.

### 2.3. Activity Measurement

The activity of *α*-L-rhamnosidase expressed by naringinase was evaluated using 0.20 mM of 4-NRP in 20 mM citrate buffer at pH 3.4, while the activity of expressed *β*-D-glucosidase was determined using 0.20 mM 4-NGP in 20 mM citrate buffer at pH 3.4. A naringinase concentration of 50 mg L^−1^ was used in these experiments. The enzymatic hydrolysis was followed spectrophotometrically. The absorption was measured every 1 min during 30 min, at 30.0°C. In both reactions 1 mol of substrate led to 1 mol of product. A calibration curve was built for each substrate and respective product. The enzyme activity (*A*) of *α*-L-rhamnosidase and *β*-D-glucosidase activities of naringinase was calculated by linear regression on the first data-points during the initial 30 min reaction time.

### 2.4. pH Profile

The influence of pH on *β*-D-glucosidase and *α*-L-rhamnosidase specific activity (*A*) can be described by

(1) $$A=\frac{A_{\max\;}}{1+10^{\text{p}K_{1}-\text{pH}}+10^{\text{pH}-\text{p}K_{2}}},$$

where *A*_max_ is the maximal enzyme-specific activity and *K*_1_, *K*_2_ are the equilibrium constants of enzyme deprotonation [19, 20].

The naringinase activity pH profiles were obtained through nonlinear regression by minimising the residual sum of squares between the experimental data points of the specific activity versus pH and those estimated by the model, using Solver add-in from Microsoft Excel 2003 for Windows XP and considering the following options: Newton method; 100 iterations, precision of 10^−6^, 5% of tolerance, and 10^−4^ convergence. The experimental optimum pH values were used as initial parameters of the nonlinear regression, and no constraints were used. Optimum pH values were then determined from the zero calculation of (1) first derivative.

### 2.5. Inactivation Kinetics

In order to study *β*-D-glucosidase and *α*-L-rhamnosidase inactivation kinetics, a temperature range of 75–85°C and a pH range of 3.19–6.01 were used. Naringinase thermal inactivation was carried out in Eppendorf tubes (1.5 mL), at isothermal conditions (±0.1°C) using a thermostatic water bath (Julabo Hc/F18). The inactivation time ranged from 2.5 to 160 min according to the temperature used. Inactivation was stopped by removing the enzyme samples to an ice water bath for 5 min. Enzyme activity was measured, in triplicate, immediately as well as one day after thermal inactivation, without occurring reactivation. *A*_0_ was the activity of the control, that is, the enzyme sample without being submitted to inactivation.

First-order inactivation rate constants and *α*_1_ parameter were determined by nonlinear regression minimising the residual sum of squares between the experimental data points of the residual activity versus time and those estimated by the model, using Solver add-in from Microsoft Excel 2003 for Windows XP, considering the following options: Newton method; 100 iterations, precision of 10^−6^, 5% tolerance, and 1 × 10^−4^ convergence. The first-order deactivation rate constant obtained form linear regression of ln *A*_*r*_ versus *t *was used as the initial values for the nonlinear regression *k*_1_ parameter. The nonlinear regression parameters were restricted to positive numbers, and also *α*_1_ was restricted to the gap between 0 and 1. 

The time needed to achieve a specific activity of *β*-D-glucosidase 0.01% of the specific activity of *α*-L-rhamnosidase was *t*_0.01%_. At these time (*t*_0.01%_) the *β*-D-glucosidase was considered completely inactivated. The *t* values were determined through extrapolation of the kinetic inactivation profiles obtained under different conditions of pH and temperature. The relative activity of *α*-L-rhamnosidase at this time values (*t*) was determined for each specific condition of pH and temperature.

### 2.6. Experimental Design

The optimized temperature and pH inactivation conditions of *β*-D-glucosidase activity of naringinase were established via Response Surface Methodology (RSM). Using this methodology two variables were tested simultaneously with a minimum number of trials, according to adequate experimental designs, which enables to find interactions between variables [21]. The experimental design methodology makes use of statistical tools for selecting a minimum set of experiments adequately distributed in the experimental region (experimental matrix). 

In this study, *β*-D-glucosidase inactivation was carried out following a central composite rotatable design (CCRD). For the design setup, three different coded levels for each factor were used, low (−1), center (0), and high (+1), as indicated in Table 1. The response variables were *β*-D-glucosidase and *α*-L-rhamnosidase activities (mg mL^−1^ min^−1^). The experiments were performed in random order. Triplicate experiments were carried out at all design points.

The choice of experimental domains resulted from preliminary studies. The hydrolysis was carried out in 20 mM citrate buffer. A total of 11 experiments were carried out in each CCRD: four factorial points (coded levels as (+1) and (−1)), four star points (coded as (+√2), and (−√2)) and three centre points (coded as 0) (Table 1).

### 2.7. Statistical Analysis

With CCRD, 5 levels for each factor were used which enabled to fit second-order polynomials to the experimental data points. The results of each CCRD were analyzed using the software “Statistic,” version 6, from StatSoft, USA. Both linear and quadratic effects of the two variables under study, as well as their interactions, on *β*-D-glucosidase and *α*-L-rhamnosidase activities were calculated. Their significance was evaluated by analysis of variance. 

Experimental data were fitted to a second-order polynomial model and the regression coefficients obtained. The generalized second-order polynomial model used in the response surface analysis was as follows:

(2) $$Y=\beta_{0}+\beta_{1}X_{1}+\beta_{2}X_{2}+\beta_{11}X_{1}^{2}+\beta_{22}X_{2}^{2}+\beta_{12}X_{1}X_{2},$$

where *β*_0_, *β*_1_, *β*_2_, *β*_11_, *β*_22_, and *β*_12_ are the regression coefficients for intercept, linear, quadratic and interaction terms, respectively, and *X*_1_ and *X*_2_ are the independent variables, temperature and pH.

The fit of the models was evaluated by the determination coefficients (*R* ^2^) and adjusted *R* ^2^ (*R*_adj_^2^).

### 2.8. Verification Experiments

Optimal conditions for the inactivation of *β*-D-glucosidase activity of naringinase keeping *α*-L-rhamnosidase with a high activity were dependent on temperature and pH conditions obtained using the predictive model equations of RSM. The experimental and predicted values were compared in order to determine the validity of the model.

### 2.9. Methods for Flavonoid Production and Purification

Prunin was obtained through the hydrolysis of a 10 mM naringin solution, using 50 mg L^−1^ of naringinase, with its *β*-D-glucosidase selectively inactivated, in 20 mM citrate buffer, pH 3.4, 60.0°C for 6 hours. Prunin precipitated after 12 hours at 4°C and was recovered through vacuum filtration. Consecutively it was dissolved in hot water and was filtered. Prunin was obtained through recrystallization from water.

Naringenin was obtained through the hydrolysis of a 10 mM naringin solution, using 50 mg L^−1^ of naringinase in 20 mM acetate buffer, pH 4.0, 60.0°C for 6 hours. Naringenin precipitated after 12 hours at 4°C and was recovered through vacuum filtration. Consecutively it was dissolved in hot ethanol and filtered. Naringenin was obtained through recrystallization from ethanol and water.

Isoquercetin was obtained through the hydrolysis of a 5 mM rutin solution, using 50 mg L^−1^ of naringinase, with *β*-D-glucosidase activity selectively inactivated, in 20 mM citrate buffer, pH 3.4, 60.0°C for 6 hours. Isoquercetin precipitated after 12 hours at 4°C and was recovered through vacuum filtration. Consecutively it was dissolved in hot ethanol and was filtered. Isoquercetin was obtained through recrystallization from ethanol and water.

Quercetin was obtained through the hydrolysis of a 5 mM rutin solution, using 50 mg L^−1^ of naringinase in 20 mM acetate buffer, pH 4.0, 60.0°C for 6 hours. Quercetin precipitated after 12 hours at 4°C and was recovered through vacuum filtration. Consecutively it was dissolved in hot ethanol and was filtered. Quercetin was obtained through recrystallization from ethanol and water.

## 3. Results and Discussion

### 3.1. pH Profile

**Figure 2:**
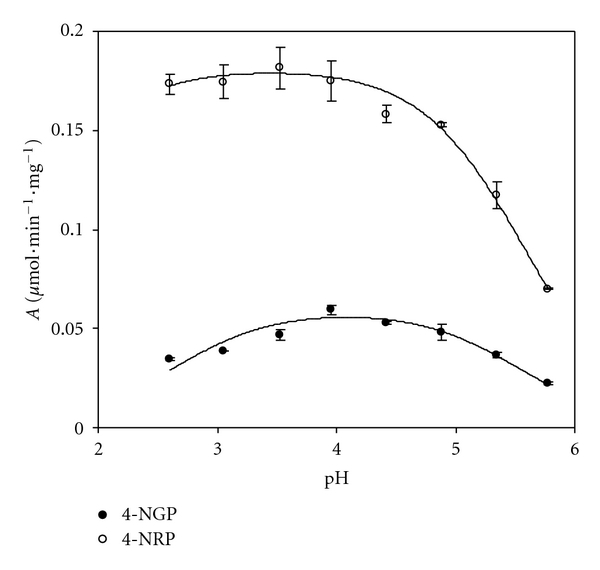
pH profile of 4-NGP and 4-NRP hydrolysis using naringinase in sodium citrate buffer 20 mM (mean value ± standard error).

The activity pH profile of both *α*-L-rhamnosidase and *β*-D-glucosidase activities of naringinase was studied hydrolysing the specific substrates, 4-NRP and 4-NGP, respectively. This pH profile was studied between 2.5 and 5.8, in citrate buffer.  Figure 2 shows the distinct activity pH profiles of *α*-L-rhamnosidase and *β*-D-glucosidase activities of naringinase. From these studies and adjusting the model of (1), the optimum pH was found to be 3.4 and 4.1, respectively, with a maximum specific activity of 0.181 and 0.060 *μ*mol mg^−1^ min^−1^, for *α*-L-rhamnosidase and *β*-D-glucosidase activities of naringinase (Table 2). Jurado et al. [19] adjusted a similar model to the experimental data of *β*-D-galactosidase activity versus pH. 

In a previous work [20] the specific activity of *β*-D-glucosidase in 20 mM acetate buffer, pH 4.0 was 0.086 *μ*mol min^−1^ mg^−1^, while in this study, in 20 mM citrate buffer, pH 4.0 it was found to be 0.060 *μ*mol min^−1^ mg^−1^. This lower *β*-D-glucosidase specific activity corresponded to a 30% activity decrease when citrate buffer was used instead of acetate. These results highlight the importance of buffer (citrate) and pH (3.4) to selectively inactivate *β*-D-glucosidase activity of naringinase. Norouzian et al. [22] observed a naringinase activity inhibition with 20 mM citric acid buffer. In further studies of *β*-D-glucosidase inactivation citrate buffer was used as bioreaction media.

### 3.2. Inactivation Kinetics

**Figure 3:**
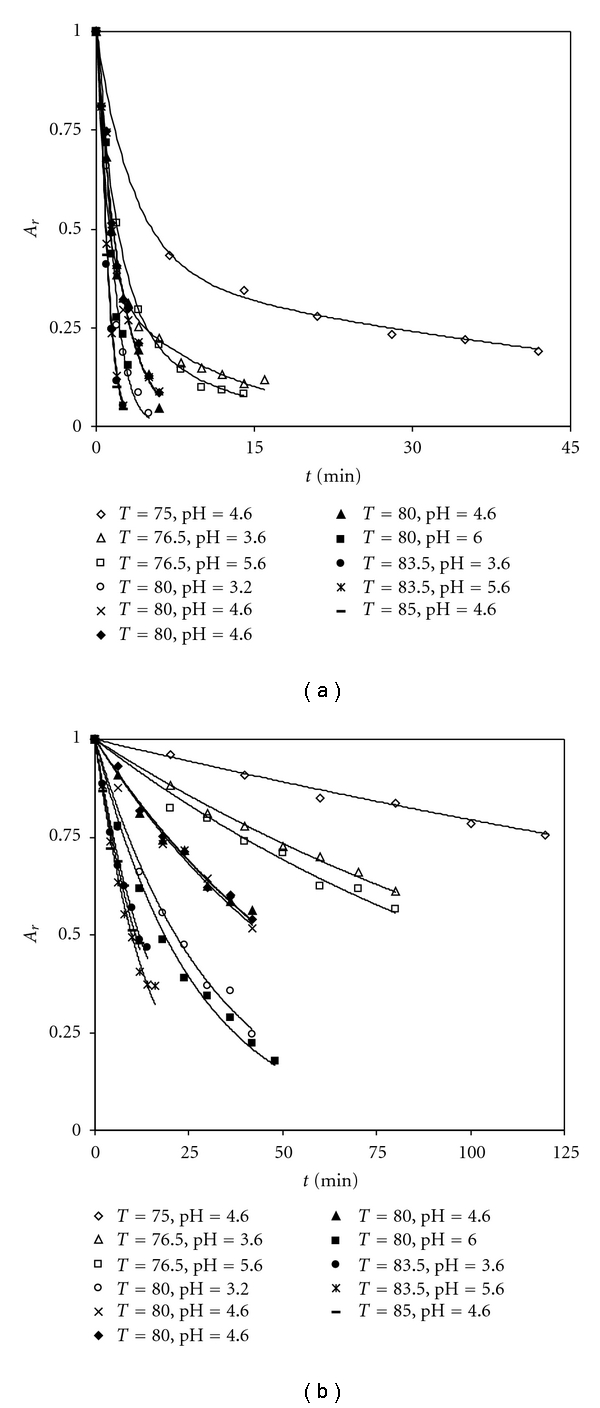
Thermal inactivation under combined temperature and pH conditions of *β*-D-glucosidase (a) and *α*-L-rhamnosidase (b) activities of naringinase.

Naringinase was inactivated using combined temperature and pH conditions, between 75.0–85.0°C and 3.2–6.0, respectively. Thus, temperature and pH on stability of *β*-D-glucosidase and *α*-L-rhamnosidase were evaluated on a minimum set of optimal selected experiments. The inactivation behaviour for *β*-D-glucosidase and *α*-L-rhamnosidase activities of naringinase was distinct from each other under the same temperature and pH conditions (Figure 3).

To describe the inactivation kinetics for *β*-D-glucosidase and *α*-L-rhamnosidase activities of naringinase, residual activity (*A*_*r*_) was defined as the ratio between the specific activity after each time inactivation period* (A) *and the specific activity without inactivation (*A*_0_).

Both pH and temperature treatments could be described adequately by a series-type enzyme inactivation model (3) involving first-order steps in an inactivation sequence as well as an active intermediate:

(3) $$A_{0}\overset{k_{1}}{\to }A_{1}\overset{k_{2}}{\to }A_{2}.$$

A biphasic inactivation nature was observed for *β*-D-glucosidase with a final state totally inactivated (*α*_2_ = 0) adjusted to (4) [23].

(4) $$A_{r}=\left[1+\frac{\alpha_{1}k_{1}}{k_{2}-k_{1}}\right]e^{-k_{1}t}-\left[\frac{\alpha_{1}k_{1}}{k_{2}-k_{1}}\right]e^{-k_{2}t}$$

*k*_1_ and *k*_2_ were the first and second deactivation rate coefficients, respectively; *A*_0_, *A*_1_, and *A*_2_ were the specific activities of the initial active enzyme, enzyme intermediate, and final enzyme state, respectively; *α*_1_ was the specific activities ratio *A*_1_ *A*_0_^−1^ and *A*_2_ *A*_0_^−1^, respectively. A first inactivation step was followed by a second one with the existence of an enzymatic intermediate having a lower specific activity than the initial enzyme native state (*α*_1_ < 1) (Table 3) and a final state where the enzyme is completely inactivated (*α*_2_ = 0) [23]. The first faster deactivation step observed may correspond to the unfolding of the carbohydrate portion, lowering its activity relative to the initial state (*α*_1_); afterwards, the second slower step may apply to the embodiment of enzyme inactivation.

On the other hand, *α*-L-rhamnosidase inactivation as well as the inactivation of *β*-D-glucosidase under the conditions of 80.0°C and pH 4.6, occurred according to the classical first-order inactivation model (Figure 3)

(5) $$A_{r}=e^{-k_{1}t}.$$

Table 3 shows the inactivation parameters determined at different temperature and pH conditions for both *β*-D-glucosidase and *α*-L-rhamnosidase.

Tsen et al. [24] and Ellenrieder and Daz [25] reported naringinase (from *Penicillium decumbens*) deactivation profiles at pH 3.5–3.7. *α*-L-Rhamnosidase from *Aspergillus terreus* [26] and *Aspergillus nidulans* [27] when incubated at pH values lower than 4.0 rapidly lost activity, whereas *Aspergillus aculeatus* [28] was shown to be insensitive to pH in the range 3–8. Comparing our results with available stability data of purified fungal *α*-L-rhamnosidases referred by different authors [26, 27, 29, 30], it can be pointed out that our developed method avoiding *α*-L-rhamnosidase purification with *β*-D-glucosidase inactivation is an effective and cheap method.

### 3.3. RSM

In this study a central composite design and response surface methodology (RSM) were applied in order to acquire pH-temperature conditions to selectively inactivate *β*-D-glucosidase activity expression from naringinase, keeping *α*-L-rhamnosidase with high activity. The experiments were carried out according to a design 2^2^ and a CCRD, as a function of both the temperature (*T*) and pH. The obtained results, *α*-L-rhamnosidase residual activity of naringinase corresponding to a *β*-D-glucosidase inactivation, were used to calculate the significant effects, either linear or quadratic, of the 4-NRP hydrolysis reaction. 

**Figure 4:**
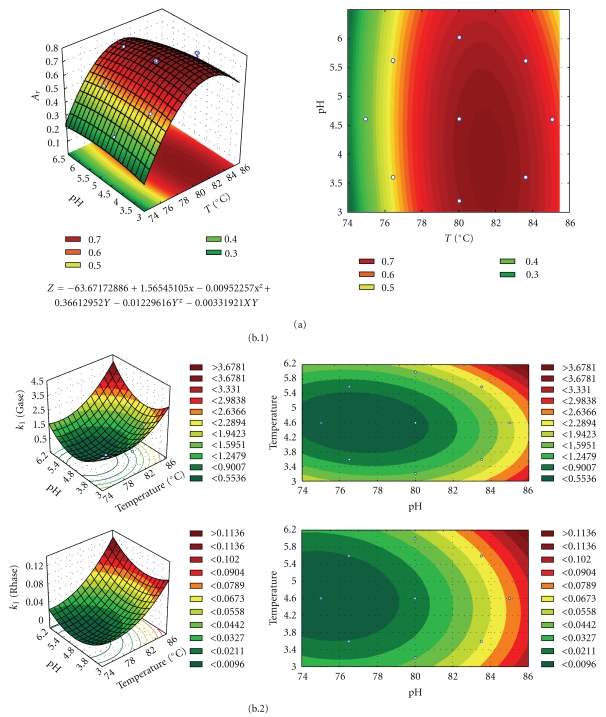
Response surfaces fitted to the experimental data points corresponding to (a) *α*-L-rhamnosidase residual activity and (b) to first deactivation rate coefficients (*k*_1_) for (b.1) *α*-L-rhamnosidase (rhase) and (b.2) *β*-D-glucosidase (gase) activities of naringinase, as a function of temperature (*T*) and pH.

The experimental results showed that *α*-L-rhamnosidase residual activity and *β*-D-glucosidase inactivation were affected by pH and temperature individually and interactively (Figure 4). In Table 4 are presented the effects and respective significant levels (*P*) of the temperature (*T*), pH, and interaction (*T* × pH) on the *α*-L-rhamnosidase residual activity. Therefore, negative effects of the factors temperature (*T*) or pH or their interaction (*T* × pH) indicate that the response decreased with the increase in these factors. Linear and quadratic terms of temperature were highly significant, *P* < 0.001, while the linear and quadratic terms of pH were significant (*P* < 0.05) for *α*-L-rhamnosidase activity (Table 4). A negative interaction between the variables tested (*T* × pH) on *α*-L-rhamnosidase activity indicated that higher activities are obtained at higher temperatures and lower pH within the experimental domain. 

In addition, RSM was also applied to the first deactivation rate coefficients (*k*_1_) for *α*-L-rhamnosidase and *β*-D-glucosidase obtained at the different experimental conditions tested (Table 3). It was not possible to apply RSM to the parameters *α*_1_ and *k*_2_, as shown in Table 3, because the models are adjusted at different conditions. 

Moreover, in Figure 4 is presented the effects of temperature and pH on the first deactivation rate coefficient (*k*_1_) for *α*-L-rhamnosidase and *β*-D-glucosidase activities of naringinase. Highly significant effects (*P* < 0.001) were obtained for *k*_1_ of *β*-D-glucosidase inactivation at different pH and temperature conditions (Table 4). The first deactivation rate coefficient (*k*_1_) of *α*-L-rhamnosidase and *β*-D-glucosidase increased with temperature and pH (Figure 4).

A least-squares technique was used to fit quadratic polynomial models and obtain multiple regression coefficients for *α*-L-rhamnosidase activity which are summarized in Table 5. Examination of these coefficients indicated that temperature effects on *α*-L-rhamnosidase activity both linear and quadratic terms were highly significant, *P* < 0.001 (Table 5). The linear and quadratic terms of pH were significant on *α*-L-rhamnosidase activity (*P* < 0.05) (Table 5). Moreover, linear and quadratic terms had high and significant effects (*P* < 0.001 and *P* < 0.05) on first deactivation rate coefficient of *α*-L-rhamnosidase, while for *β*-D-glucosidase significant effects are presented (*P* < 0.01 and *P* < 0.05) (Table 5).

Therefore, curved surfaces were fitted to the experimental data (Figure 4). Partial differentiation of these polynomial equations was used to find the optimum points, that is, the stationary points. The least-square estimates of the coefficients of the model were calculated from the values of the response for each experiment in the chosen experimental matrix. The relationships between independent and dependent variables in the three-dimensional representations are convex surfaces, for *α*-L-rhamnosidase activity (Figure 4). The obtained response surface (Figure 4) was described by second-order polynomial equations to the experimental data points, as a function of temperature and pH (Table 5). In the design of these models, the significant effects (*P* < 0.05) and those that presented a confident range smaller than the value of the effect or smaller than the standard deviation were included in these model equations. In fact, these later effects have a lower probability, but their values are not small enough to be neglected.

The high values of *R* ^2^ and *R*_adj_^2^ of the model (Table 5) showed a close agreement between the experimental results and the theoretical values predicted by the model [10]. The adjusted coefficients of determination for *α*-L-rhamnosidase activity (*R*_adj_^2^ = 0.936) implied that 93.6% of the variations could be explained by the fitted model. 

The ANOVA for the two response variables (temperature and pH) indicated that the model developed for *α*-L-rhamnosidase activity was adequate with the linear and the quadratic term with high significant effect (<0.05%) (data not showed).

The regression models allowed the prediction of the effects of the two parameters, temperature and pH on *α*-L-rhamnosidase activity and *β*-D-glucosidase inactivation. These optimal conditions were a temperature of 81.5°C and pH 3.9 (Table 6). 

 Once tested, the model may be used to predict the value of the response(s) under any conditions within the experimental region. 

These results showed how naringinase can be used to selectively catalyze reactions like glycosides hydrolysis towards monoglycosylated flavonoids.

### 3.4. Verification of the Optimal Temperature and pH Inactivation Conditions

The optimal conditions of temperature and pH found using RSM (Table 6) were tested experimentally in order to confirm the predicted results. 

**Figure 5:**
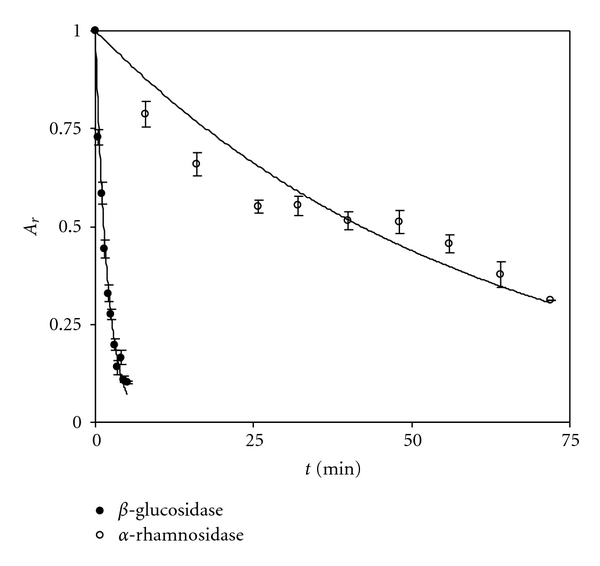
Inactivation kinetics of *β*-D-glucosidase and *α*-L-rhamnosidase activities of naringinase at 81.5°C and pH 3.9.

Figure 5 shows the inactivation profiles of both *β*-D-glucosidase and *α*-L-rhamnosidase under 81.5°C and pH 3.9. Moreover, the verification experiments proved that the predicted values for *α*-L-rhamnosidase residual activity (0.77) for the model was satisfactorily achieved within more than 95% confidence interval. The time needed for *β*-D-glucosidase activity to reach 0.01% of *α*-L-rhamnosidase activity was determined through extrapolation of *β*-D-glucosidase inactivation and corresponded to 15 minutes and 26 seconds. At this time the *α*-L-rhamnosidase residual activity was 0.78 which is quite similar to the value predicted with RSM (Table 6).

### 3.5. Production and Identification of Bioactive Compounds

**Figure 6:**
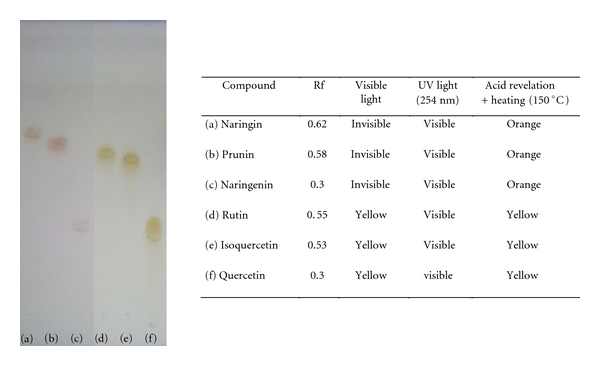
TLC of (a) naringin (Rf = 0.62), (b) prunin (Rf = 0.58), (c) naringenin (Rf = 0.30), (d) rutin (Rf = 0.55), (e) isoquercetin (Rf = 0.53), and (f) quercetin (Rf = 0.30). All of compounds were visible with UV light (254 nm). After acid revelation + heating (150°C), naringin, prunin, and naringenin showed orange spots and rutin, quercetin, and isoquercetin, yellow.

Once *β*-D-glucosidase of naringinase was selectively inactivated, the residual *α*-L-rhamnosidase activity was used for the production of flavonoids glycosides starting from rutinosides, (c.f. Section 2.9) (Figure 6). Adequate purification procedures were used, and compounds identification was carried out through HPLC LC-MS analysis.

Naringin enzymatic hydrolysis lead to prunin, a very expensive product. Isoquercetin was obtained from rutin enzymatic hydrolysis in a production yield of 61%. 

The aglycones were also produced from rutinosides using native naringinase, expressing *α*-L-rhamnosidase and *β*-D-glucosidase. As long as a sugar moiety was removed from the rutinoside to the aglycone, a polarity decrease was observed, as shown in Figure 6. Naringenin was obtained with a production yield of 49% from naringin, while quercetin was obtained from rutin (Figure 2) in a yield of 86%. 

These outcomes showed the high potential of the developed method on the production of monoglycosylated flavonoids.

## 4. Conclusions

The inactivation of *β*-D-glucosidase activity of naringinase was affected by pH and temperature individually and interactively, in citrate buffer. This inactivation could be described by response surfaces that enabled to fit second-order polynomials equations. A closed agreement between the experimental *α*-L-rhamnosidase residual activity (0.78) and the predicted value by the model (0.77) made RSM an appropriate tool to achieve temperature and pH optimized values for the selective inactivation of *β*-D-glucosidase activity of naringinase, at 81.5°C, pH 3.9 for 16 minutes. 

These are high innovative and sounding results showing the potential of the efficient and cheap developed method for the production of flavonoid glycosides starting from rutinosides.

## Figures and Tables

**Table 1:** Coded and decoded levels of the experimental factors used in experimental design.

CCRD	pH	Temperature (°C)
−√2	3.2	75.0
−1	3.6	76.5
0	4.6	80.0
+1	5.6	83.5
+√2	6.0	85.0

**Table 2:** Optimum pH values for the hydrolysis of 4-NGP and 4-NRP with *β*-D-glucosidase and *α*-L-rhamnosidase activities of naringinase.

Substrate	Optimum pH	Nonlinear parameters	*r* ^2^
4-NGP	4.1	*A*_max_ = 0.060, p*K*_1_ = 2.6, p*K*_2_ = 5.5	0.892
4-NRP	3.4	*A*_max_ = 0.181, p*K*_1_ = 1.3, p*K*_2_ = 5.6	0.984

**Table 3:** Thermal inactivation parameters of *β*-D-glucosidase and *α*-L-rhamnosidase activities of naringinase, under combined temperature and pH conditions.

*T* (°C)	pH	*β*-D-glucosidase				*α*-L-rhamnosidase		*A*_*r*_ (*α*-L-rhamnosidase)
		*α*_1_	*k*_1_ (*h* ^−1^)	*k*_2_	*r* ^2^	*k*_1_ (*h* ^−1^)	*r* ^2^	
75.0	4.6	0.376	0.283	0.017	0.999	0.0023	0.990	0.33
76.5	3.6	0.327	0.990	0.084	0.997	0.0062	0.994	0.57
76.5	5.6	0.226	0.486	0.093	0.999	0.0073	0.980	0.56
80.0	3.2	—	0.579	—	0.971	0.0323	0.989	0.73
80.0	4.6	—	0.428	—	0.980	0.0154	0.979	0.74
80.0	4.6	—	0.434	—	0.990	0.0149	0.991	0.75
80.0	4.6	—	0.431	—	0.992	0.0149	0.996	0.75
80.0	6.0	—	0.547	—	0.953	0.0375	0.995	0.71
83.5	3.6	—	0.869	—	0.987	0.0589	0.989	0.71
83.5	5.6	—	0.838	—	0.953	0.0711	0.991	0.65
85.0	4.6	—	0.868	—	0.997	0.0646	0.979	0.68

**Table 4:** Effects and respective significance levels (*P*) of temperature (*T*) and pH on *α*-L-rhamnosidase residual activity (*A*_*r*_) and on first deactivation rate coefficient (*k*_1_) for *α*-L-rhamnosidase and *β*-D-glucosidase activities of naringinase.

Variable	*A*_*r*_	*k*_1_	*k*_1_
	(*α*-L-rhamnosidase)	(*α*-L-rhamnosidase)	(*β*-D-glucosidase)
*T* (linear term)	18.1467***	0.0509***	1.0856***
*T* (quadratic term)	−23.8667***	0.0188**	0.6455***
pH (linear term)	−2.5064*	0.0052	0.1745***
pH (quadratic term)	−2.4592*	0.0211**	0.9921***
*T* × pH	−2.3500	0.0055	0.223***

*P < 0.05; **P < 0.01; ***P < 0.001.

**Table 5:** Second-order model equations for the response surfaces fitted to the experimental data points of *α*-L-rhamnosidase residual activity (*A*_*r*_), on first deactivation rate coefficient (*k*_1_) for *α*-L-rhamnosidase and *β*-D-glucosidase activities of naringinase, as a function of temperature (*T*) and pH, and respective *R* ^2^ and *R*_adj_^2^.

Coefficient	*A*_*r*_	*P*	*k*_1_	*P*	*k*_1_	*P*
	(*α*-L-rhamnosidase)		(*α*-L-rhamnosidase)		(*β*-D-glucosidase)	
*β*_0_	−63.6717	0.0004	4.8541	0.0068	178.4516	0.0419
Linear						
*β*_1_	1.5645	0.0004	−0.1193	0.0064	−4.2067	0.0456
*β*_2_	0.3661	0.0322	−0.1579	0.0436	−7.0252	0.1032
Quadratic						
*β*_11_	−0.0095	0.0004	0.00077	0.0055	0.0263	0.0443
*β*_22_	−0.0123	0.0355	0.01056	0.0039	0.4961	0.0106
Cross-product						
*β*_12_	*─* 0.0033	0.0530	0.00079	0.3056	0.03186	0.4793
*R* ^2^	0.936	—	0.9812	—	0.9047	—
*R*_adj_^2^	0.872	—	0.9624	—	0.8095	—

**Table 6:** Verification of the developed model for *β*-D-glucosidase inactivation at optimal temperature and pH conditions leading the best *α*-L-rhamnosidase residual activity of naringinase.

	Predicted	Experimental
Temperature (°C)	81.5	81.5
pH	3.9	3.9
Time (min)	16	16
*α*-L-rhamnosidase residual activity	0.77	0.78
*β*-D-glucosidase residual activity	0.0001	0.0001
